# Intelligent Warehouse in Industry 4.0—Systematic Literature Review

**DOI:** 10.3390/s23084105

**Published:** 2023-04-19

**Authors:** Agnieszka A. Tubis, Juni Rohman

**Affiliations:** Faculty of Mechanical Engineering, Wroclaw University of Science and Technology, 50-370 Wroclaw, Poland; juni.rohman@pwr.edu.pl

**Keywords:** Warehouse 4.0, Industry 4.0, literature review, PRISMA, classification framework

## Abstract

The development of Industry 4.0 (I4.0) and the digitization and automation of manufacturing processes have created a demand for designing smart warehouses to support manufacturing processes. Warehousing is one of the fundamental processes in the supply chain, and is responsible for handling inventory. Efficient execution of warehouse operations often determines the effectiveness of realized goods flows. Therefore, digitization and its use in exchanging information between partners, especially real-time inventory levels, is critical. For this reason, the digital solutions of Industry 4.0 have quickly found application in internal logistics processes and enabled the design of smart warehouses, also known as Warehouse 4.0. The purpose of this article is to present the results of the conducted review of publications on the design and operation of warehouses using the concepts of Industry 4.0. A total of 249 documents from the last 5 years were accepted for analysis. Publications were searched for in the Web of Science database using the PRISMA method. The article presents in detail the research methodology and the results of the biometric analysis. Based on the results, a two-level classification framework was proposed, which includes 10 primary categories and 24 subcategories. Each of the distinguished categories was characterized based on the analyzed publications. It should be noted that in most of these studies, the authors’ attention primarily focused on the implementation of (1) Industry 4.0 technological solutions, such as IoT, augmented reality, RFID, visual technology, and other emerging technologies; and (2) autonomous and automated vehicles in warehouse operations processes. Critical analysis of the literature also allowed us to identify the current research gaps, which will be the subject of further research by the authors.

## 1. Introduction

The main goal of any supply chain management is to achieve coordination and linkages between all processes and cooperating entities [1]. The impact of information exchange on the development of supply chains has become more significant with advances in Information Technology (IT) [1]. Lotfi et al. [2] note explicitly that with advances in information technology, different network structures can be modeled to make the coordination within supply chain partners even closer. In addition, increasing the amount and scope of information exchanged reduces uncertainty. As a result, customers receive a higher quality product at a lower cost in a shorter period. A significant trend aimed at improving information flows in supply chains is the digital transformation observed for several years. In the initial phase of the transformation, digitization was equated with an electronic version of a document or sound [3]. However, the process is now seen from a broader perspective and is identified with the various sociotechnical phenomena and processes of adopting and using digital technologies in a broader individual, organizational, and societal context [4]. Indeed, digitization introduces changes in supply chain operations that affect equally [5] (1) the organization’s technical capabilities and technological infrastructure, (2) the organization’s strategies and directions, (3) the potential of the human factor, and (4) the integration of all stakeholders around the organization. Digitization also supports the integration of management structures [6] and increases the transparency and traceability of supply chain processes [7]. Therefore, its implementation is usually focused mainly on the use of multiple techniques that monitor real-time items, reduce the idle time in production, visualize a smart interconnected network, make more efficient use of resources, optimize supply chain inventories to enable supplier risk assessment, and provide excellent visibility along the supply chain [8].

Digitization of logistics processes enables companies to monitor real-time material flows and handle units better. However, the application of new technologies in logistics should be based on the following four principles [9]:

- Decision support and decision-making—this refers to the potential of artificial intelligence and big data analysis to automate decision-making processes or support human decisions using a data-based approach;
- Identification and interconnectivity—this refers to IoT (Internet of Things) technologies and intelligent sensors that are able to unambiguously identify products and materials and improve product tracking inside and outside companies, including intercommunication;
- Information flow—this refers to the integration of IT systems (vertical integration), which also uses cloud computing to provide access to data from multiple sources in real time to better respond to real-time production planning;
- Automation, robotics, and new production technologies—introduction of new equipment and intelligent transportation systems capable of replacing or duplicating human labor in manual activities.

Integrating physical logistics processes with digital data and increasing the visibility of each link throughout the supply chain are forcing the transformation from traditional to digital supply chains [8]. However, implementing Industry 4.0 (I4.0) solutions in manufacturing processes has naturally accelerated the digital transformation, primarily in internal logistics processes, particularly in the material and finished goods warehouse handling processes. This is because these processes significantly affect the efficiency [10] and continuity of manufacturing processes and are of dominant importance for the operation of automated production [1].

Warehousing is one of the fundamental processes implemented in supply chain management. According to [1], it is an essential part of the integration of all operations in supply chains. It is considered an essential part of product flows due to its involvement in achieving optimum and continuous operation of the production and distribution processes. According to [11], warehousing has been playing a new role for several years and can make all chain processes better integrated, not only in storing goods but also in providing better service visibility. Through this function, it becomes possible to avoid overstocking throughout the supply chain. Therefore, digitization and its use in exchanging information between partners, especially real-time inventory levels, is critical. For this reason, the digital solutions of Industry 4.0 have quickly found their way into internal logistics processes and enabled the design of intelligent warehouses, also known as Warehouse 4.0. These warehouses are designed and function following the basic principles of I4.0, which include, in particular, interoperability, virtualization, decentralization, real time, service orientation, modularity, and reconfigurability [12]. These warehouses address the current need to effectively manage all warehouse processes and design a dynamic warehouse facility that is easily accessible [13].

Changes related to the digitization and automation of warehouse operations and the changing role of the warehouse in effective supply chain management increase the demand for research in this area. This is confirmed by the growing number of recent publications related to this topic. This observed upward trend was also confirmed in the studies presented in this article (in Section 3). For this reason, there is a need to identify the dominant research areas related to the design and operation of Warehouse 4.0 and to organize (classify) the increasing number of publications.

Therefore, it is reasonable to conduct a literature review aimed at finding answers to the following research questions:

- Q1: What are the research directions related to the adaptation of warehouses to the needs of Industry 4.0 and digital supply chains over the last five years (2018–2022)?
- Q2: Which research areas are particularly interesting to the scientific community (high publication rate), and which are still in the early stages of development or less popular?
- Q3: Is there a research gap that should be analyzed, particularly in connection with the digitization and automation of warehouse processes?

Therefore, the article aims to present the results of the literature review from 2018–2022, which was the basis for identifying research areas related to Warehouse 4.0 and defining the current research gap. The presented literature review, in addition to the answers to the research questions posed above, makes an essential scientific contribution in the form of:

- Development of a two-level classification framework for research from the analyzed area according to the assumptions of the concept map;
- Conducting the qualification procedure following the adopted distribution criteria based on the results of the literature research covering 220 articles from the last five years;
- Detailed characteristics of research trends described in articles belonging to the 10 highlighted primary categories.

**Figure 1:**
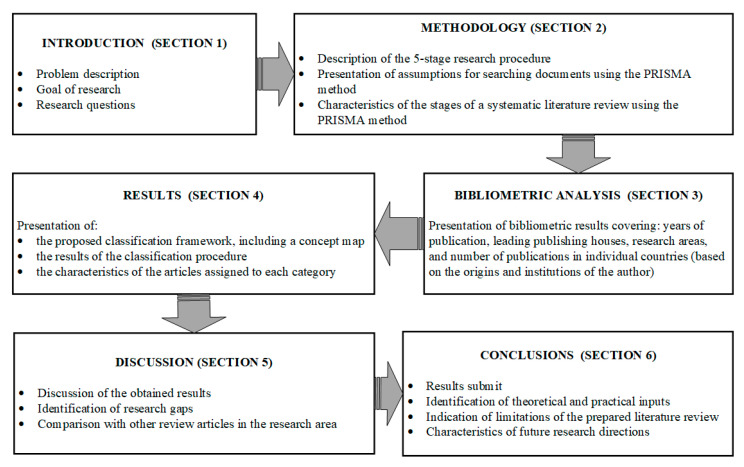
Structure of the article.

The structure of the article is shown in Figure 1.

## 2. Methodology

The choice of method for implementing the literature review depends on the research objectives set at the initial stage. The systematic literature review method was chosen for the research due to the research questions that were posed in the introduction and the related purpose of the article. According to the research presented in [14], this is the most effective method of logical exploration of the current state of knowledge and development of existing scientific knowledge on a given topic The choice was also determined because it is considered the gold standard among review methods [15]. Many authors recommend this method to identify, collect, and classify related studies in a more structured, nuanced, and reproducible manner (among others [16]). Thanks to its application, the conducted research procedure will identify all empirical evidence that fits the prespecified inclusion criteria to answer a particular research question [17].

**Figure 2:**
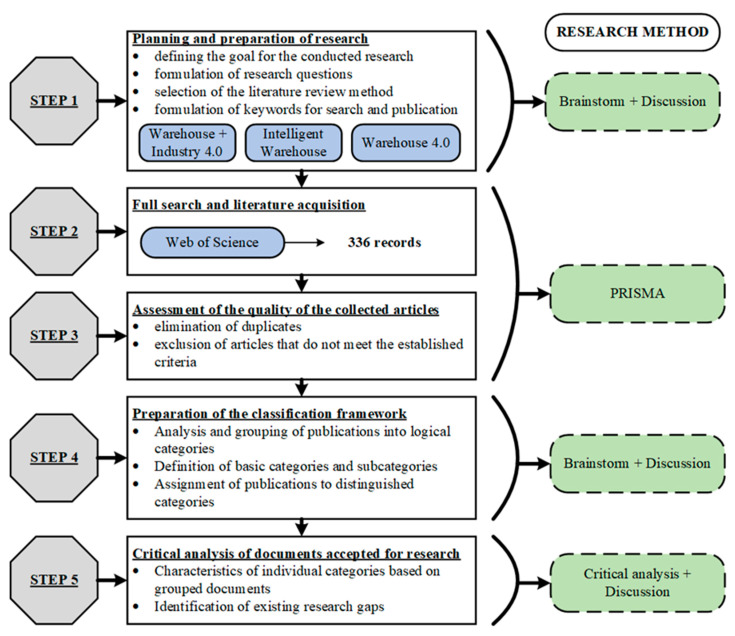
Stages of the research procedure.

The entire research procedure carried out for the article included five stages of the investigation. A detailed characterization of the steps taken in each stage is shown in Figure 2.

**Figure 3:**
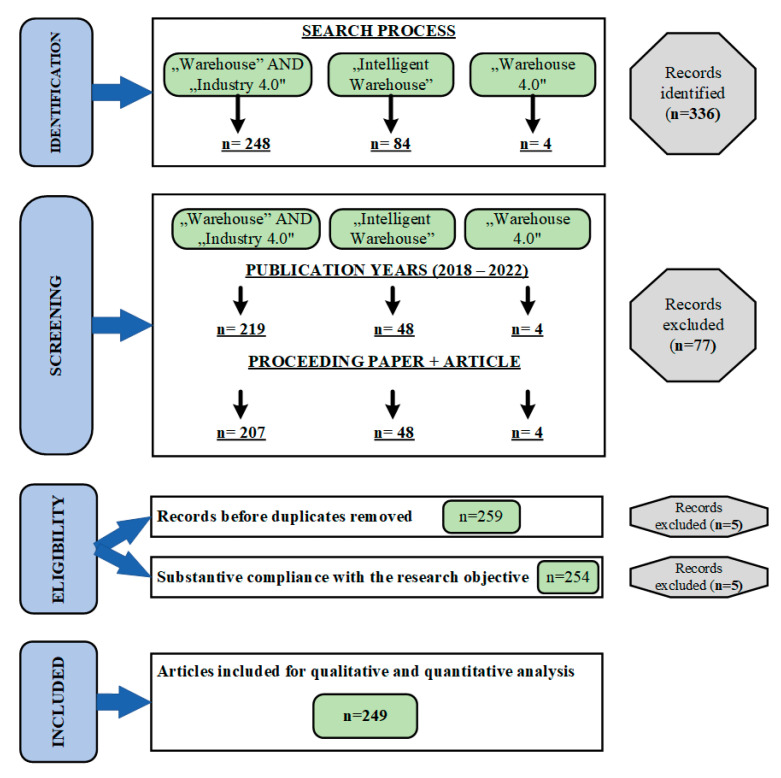
PRISMA—Flow diagram of the systematic selection literature in the analyzed research area.

The research procedure used the Preferred Reporting Items for Systematic Reviews and Meta-Analyses (PRISMA) and the Four-Phase Flow Diagram, which allows us to create a systematic and unambiguous review with established methods for identification, selection, and evaluation [18]. The entire procedure consists of four stages: identification, screening, qualification, and inclusion. The results of the procedure carried out are shown in Figure 3.

### 2.1. Identification

The database chosen for the study was the Web of Science, the most significant technical research repository typically used in literature reviews [19]. This database was also chosen because of the high quality of the documents that are recorded in it. Identification of publications was carried out through three independent searches that included the following sets of keywords:

- “warehouse” AND “industry 4.0”;
- “intelligent warehouse”;
- “warehouse 4.0”.

An analysis of review articles preceded the selection of the keywords used (among others [20,21,22]), which identified the most commonly used terms for warehouses designed and operated following the Industry 4.0 concept. The keywords were searched under “Topic”, which includes title, abstract, author’s keywords, and Keywords Plus. 

After this first stage of the PRISMA method, 336 documents were selected for the screening stage.

### 2.2. Screening

In the second stage, the screening was carried out according to the adopted inclusion criteria of the documents for further analysis. The first inclusion criterion was the date of publication of the document. The period of 1 January 2018 to 31 December 2022 was adopted as the primary qualification dates. This period was chosen because, since 2017, development of the Industry 4.0 concept and related changes in the design and operation of warehouses have been observed. The second criterion included documents registered as proceeding papers or articles in the study. Both of the indicated criteria made it possible to limit the document base to publications reflecting the latest scientific research results and, at the same time, meet the requirements of peer-reviewed publications proving the quality of the presented results.

### 2.3. Eligibility

The third stage of the procedure aims to eliminate records that do not meet the substantive and qualitative requirements for the analysis being carried out. In this case, records that occurred multiple times (and that were repeated in at least two initial searches) were eliminated at this stage. The second exclusion criterion was thematic compatibility assessed based on the published contents of the documents. In the case of five documents, the content of the abstract did not confirm thematic compliance and access to the full text was limited. This ruled out the possibility of verifying substantive compliance and qualification proceedings thoroughly.

### 2.4. Included 

Based on the identification, inclusion, and exclusion procedure thus carried out for publications related to magazines in Industry 4.0, 249 records were accepted for analysis. In the subsequent stages of the research, these documents became the basis for developing a two-level publication qualification framework for the analyzed area.

## 3. Bibliometric Analysis

**Figure 4:**
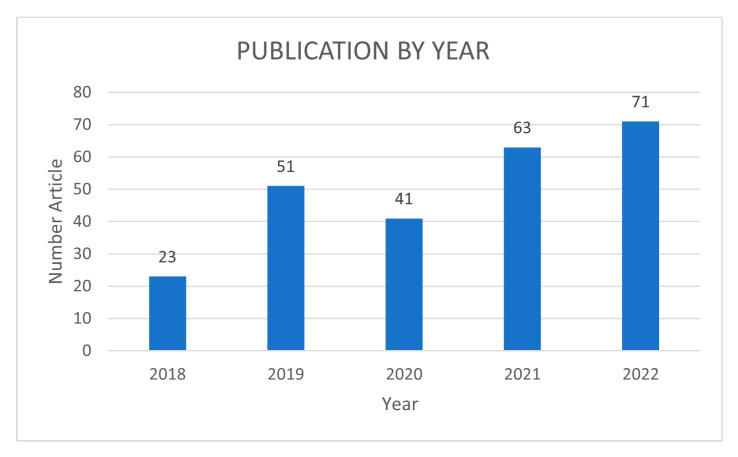
Publication by year.

A bibliometric analysis was prepared for the 249 documents accepted for analysis. This study makes it possible to present the results of the conducted literature review in quantitative and qualitative sets describing the analyzed documents. Due to the requirements introduced for the publication period of the documents accepted for analysis, it is crucial to assess the trends associated with the number of publications appearing each year (Figure 4). As can be seen, there is a clear upward trend in the analyzed period and an increasing number of publications from period to period. However, while the increase in the number of documents in 2019 relative to 2018 has more than doubled, this upward trend flattens out in subsequent periods. Given the continued development of technologies related to Industry 4.0 and the accompanying digital transformation, the observed upward trend will also continue in the next few years. The novelty of our research is the analysis of the global approach to Warehouse 4.0 also including publications that appeared by the end of 2022. So this is the current state of knowledge as of the beginning of 2023, which can be referred to in subsequent updates.

**Figure 5:**
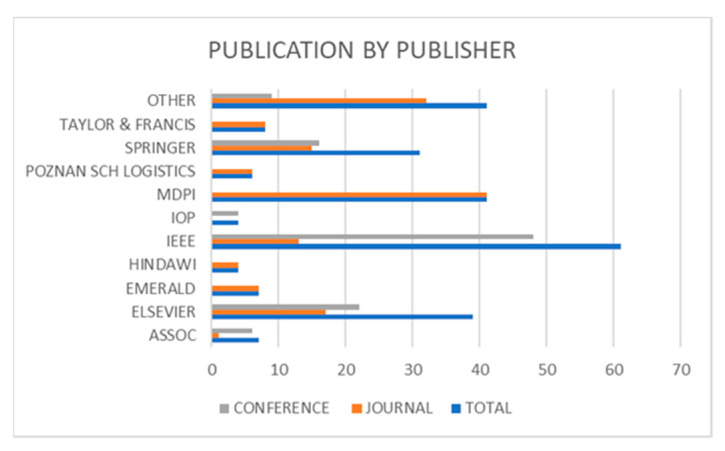
Publication by the publisher.

The number of documents published by the largest publishing houses is shown in Figure 5. The leading publisher is IEEE, with 61 publications (including 13 articles and 48 conference papers). This is understandable, as this publishing house is a leader in innovative technologies and engineering solutions publications. At the same time, it publishes conference materials from leading international conferences where the latest development trends are presented. In second place was the MDPI publishing house, with a total number of 41 publications. This publishing house has many journals related to modern technologies and their implementation in technical systems. For this reason, many researchers publish their research results there due to the faster review process than in other journals. This is important for publications on the latest technological solutions, which new ones quickly replace. Elsevier ranked third with 39 publications (13 articles and 48 conference papers).

Table 1 shows the publication research area related to warehouse and industry 4.0. The most significant number of articles came from journals and conferences related to automation and control systems—58 articles; business and economics—55 articles; and computer science—30 articles. Most were related to emerging technologies that influenced warehouse development, such as IoT, RFID (Radio-Frequency Identification), digital twins, machine learning, artificial intelligence, mixed reality, etc. However, eight articles and conference papers also came from the operation research and management field, as warehouse activities are inseparable from logistic processes. 

**Figure 6:**
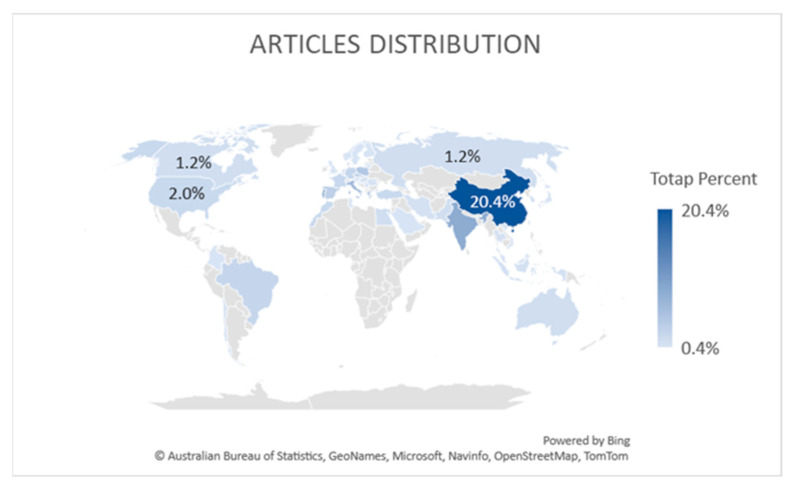
Article distribution.

An analysis of the authors’ origins and institutions allows us to map the publications we obtain by region with 50 countries in total. Most of the publications were prepared by authors from China. It should therefore be pointed out that publications from this region account for 20.4% of all analyzed articles; India—8.4%; Portugal—7.6%; Italy—6.4%; Poland—4.4%; Czech Republic and Greece—3.6% each; Spain—3.2%; Brazil and Germany with 2.0% each; France, Hong Kong, Morocco, Turkey, and the US with 2% each. Therefore, those countries are under 2.0% each. This is shown in Figure 6.

## 4. Results

**Figure 7:**
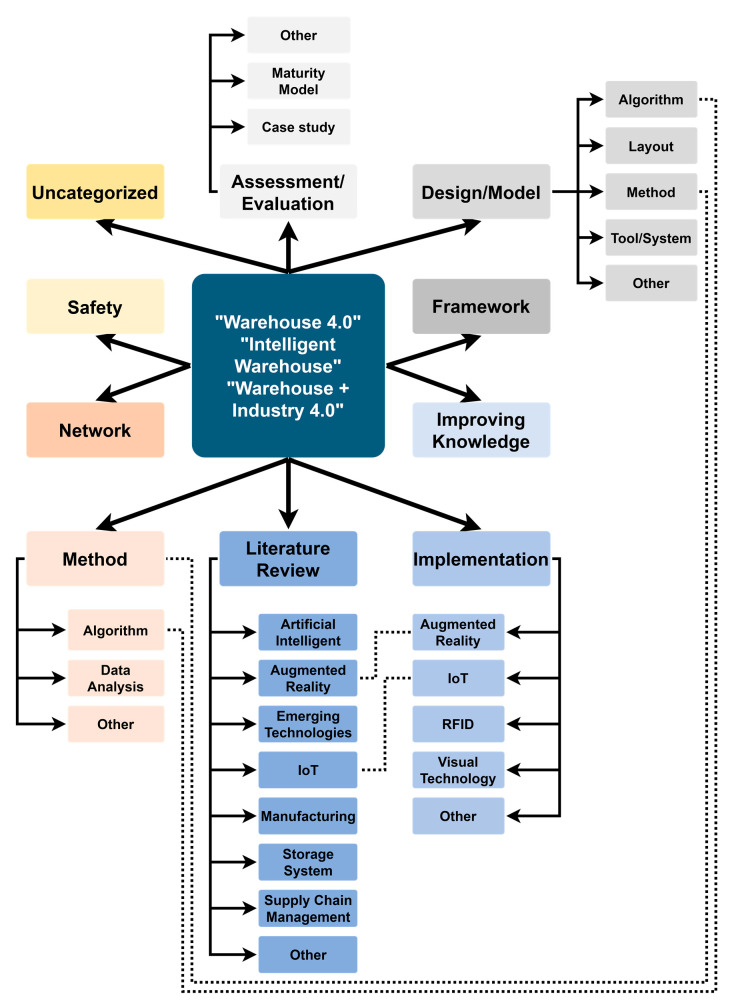
Category mapping of articles.

After analysis, the total number of articles related to Warehouse 4.0, intelligent warehouse, and Industry 4.0 obtained was 249 articles. Based on the analysis of these publications, a new classification framework for research related to Warehouse 4.0 was developed. The proposed classification distinguishes 10 primary categories, of which 5 categories are additionally divided into 24 subcategories. When the number of publications in the primary category was small, or the subject matter was very diverse, no subcategories were created in this case. Some subcategories could exist in multiple primary categories due to the interpenetration of specific thematic groups. However, an article that could not be categorized into the distinguished category was assigned to the ‘*uncategorized*’ category. All basic categories and subcategories are mapped in Figure 7 and are presented in Table S1 in the Supplementary Materials.

### 4.1. Literature Review

The literature review is an essential part of the research, collecting key sources and discussing those to gain insight into the state of the art. The literature review commonly presents challenges, solutions, benefits, and tools in a general way. Based on the findings and analyzed article, 49 articles were categorized in this category, divided into 8 subcategories based on the focus topic presented by authors. Those subcategories are:

1. Artificial Intelligence (AI) [23,24,25];
2. Augmented reality (AR) [26,27,28,29];
3. Emerging technologies as a major discussion [30,31,32,33,34,35];
4. Internet of Things (IoT) [36,37,38,39];
5. Manufacturing related with warehousing [40,41,42];
6. Storage system [43,44,45];
7. Supply chain management [46,47,48,49];
8. Other topics that covered research related to:

- Current state of discrete event simulation and digital twins [50];
- Improvement in industry 4.0 for business process [51];
- Wireless communication behavior in warehouse [52];
- Material handling [53];
- 5G in digital supply chain [54];
- Impact of industry 4.0 on logistics [55];
- Automated logistic system [56];
- Technology related to industry 4.0 for safety [57];
- Production logistic and human–computer interaction [58];
- Mixed reality in intralogistics [59];
- Using CPS (cyber-physical system) for smart warehouse [60]
- Overview of the risk value in logistics [61];
- Design of intelligent warehouse management [20];
- Spare parts and logistics management [62];
- Intelligent warehouse stocking system [21];
- Smart factory [63];
- Issue of port logistics and developing conceptual framework [64];
- Application blockchain technology [65];
- Implementation AGV (Automated Guided Vehicle) related risk analysis [66];
- Identifying challenge and strategy related smart warehouse [67]; and asset interoperability [68];
- Summarizing discussion at conference on emerging technology and factory automation about distributed warehousing and localized kitting systems [69].

### 4.2. Assessment/Evaluation

Assessment or evaluation is a method for reviewing something, which is then used as a benchmark to improve performance, a work result, or a process that is more efficient in the future. Based on the analyzed articles’ findings, 19 articles were found in this category. After further study, these articles can be categorized into subcategories. The first subcategory is *case studies.* Articles from this group present an assessment or performance evaluation of the use of emerging technologies [70,71,72,73,74,75,76] or articles that discuss a work process of Warehouse 4.0 [77,78,79,80,81]. The second subcategory is *the maturity model*. In this subcategory, the article discussed assessing a readiness model in the implementation of Warehouse 4.0. Articles that fall into this subcategory are [82,83,84,85,86]. The last subcategory is *other*. Some articles presented validation [87], testbed [88], or tool/system evaluation [89] applied to the system of Warehouse 4.0. 

### 4.3. Design/Model

The model is the basis of the designed tool or system. It can have a physical form but also intangible, e.g., a process flow diagram or mathematical formula. In this category, 43 articles were categorized and divided into 5 subcategories based on analysis. The first subcategory is *algorithm*. In these articles, the authors designed a new or developed an existing algorithm/model relating to warehouse operation in the concept of Industry 4.0. Papers in this subcategory describe optimization algorithms relating to:

- Dijkstra approach [90];
- Mathematical model for cloud-based drone routing problem [91];
- Mathematical modeling of cross-docking based on MVA for AGV [92];
- Defining linear programing model for decision support [93];
- Graph-based context tier model for flexible production system [94], and designing SBS/RS using ML-based algorithm [95].
- Designing MCDM (multiple-criteria decision model) for evaluating ERP software in warehouse and inventory management [96];
- Model predictive control of multiple AGV fault tolerance for increasing the agility [97], and developing a deep neural network for optimization transmission power of AGV [98].

The second subcategory is *layout*. In examining issues about layout warehouse, the authors focus their attention primarily on the design of the warehouse environment so that the use of emerging technologies can be supported. Studies in this subcategory include:

- Creating an environment for rapid prototyping robotic [99];
- Designing of intelligent warehouse based on tracking goods and dynamic data on a real-time basis [100];
- Layout design process for automated warehouse [101], or new plant based on industry 4.0 [102].

The third subcategory is *tool/system*. This category includes studies in which the authors designed:

- An automated machine sweeper [103];
- Smart counting for unboxed stock [104];
- Collaborative robot and AGV [105], robot for industry 4.0 [106], health and safety inspection autonomously robot for detecting hazard event [107], mechatronic interface for mobile robot [108];
- Lean Value Stream Mapping 4.0 tool for logistic process [109];
- Novel shuttle for picking system [110];
- StoreMe-Mr for intelligent warehouse control [111];
- Software framework of IoT [112];
- Indoor positioning system [113];
- Intelligent logistics warehousing and handling robot from mechanical perspective [114];
- Comprehensive monitoring system for intelligent warehouse [115];
- Architecture for developing smart warehouse [22];
- Warehouse management system using MySQL [116];
- Automating cross-docking system [117];
- Indoor UAV (Unmanned Aerial Vehicle) equipped with an onboard autonomous navigation system [118].

This subcategory also includes studies in which the authors presented models of tools or systems supporting work in the warehouse. This group includes studies on:

- Intelligent warehouse monitoring model using distributed system and edge computing [119];
- Modeled task assignment model of automation for scheduling technology [120];
- Modeling robot automatic task [121];
- Modeling UAV interconnection mechanism [122];
- Modeling intelligent software for warehouse management [123];
- Creating robot communication model in ROS [124];
- Dynamic model warehouse automation [125];
- Improving position measurement and corresponding path planning of AGV guided using visual sensor [126];
- Build a model for implementation of logistics 5.0 [127].

The last subcategory is *other.* This subcategory includes a publication whose authors presented a general model that could not be assigned to the distinguished subcategories. However, these publications were related to Warehouse 4.0 as they were concerned with modeling mixed assembly line [128], and modeling smart factory based on Industry 4.0 [129].

### 4.4. Framework

A framework is a proposal of assumptions for a system or an organizational concept formulated as part of the conducted research. Its task is to present the general mechanisms of operation and identify the key elements/components that make up this system. Many researchers describe general assumptions for the system/organizational solution, which are then adapted to the needs of the client and the specificity of the functioning of a given warehouse. The analyzed publications referred to the conceptual framework for:

- Digital Twin for industry automated system [130];
- Distributed semantic for collaborative robot [131];
- For intelligent automation [132], and autonomous robot [133];
- Agent-oriented smart factory for problem and domain definition AOSR (agent-oriented storage and retrieval) in warehouse [134];
- Of warehouse resource management system based on Industry 4.0-driven technologies [135], and IoT for warehousing [136];
- For assessment of sustainable warehousing [137], and for a smart and sustainable supply chain [138];
- Fault-tolerant design for forklift [139];
- Of order picking 4.0 concept [140];
- For new logistic center [141], and logistics 4.0 concept [12].

### 4.5. Implementation

The biggest challenge in the development of intelligent warehouses is the implementation of Industry 4.0 solutions. This implementation requires infrastructural and organizational changes and mental changes in some cases. The results of research indicate that this topic has been critical in recent years and often appears in published documents from the area studied. Considering the number of publications on implementing each technology, it is reasonable to divide them into leading and other emerging technologies. Articles belonging to each group are presented in Table 2.

### 4.6. Improving Knowledge

Along with the changing era and the development of existing technology, humans must continuously improve their knowledge and skills. Moreover, with the era shifting from traditional warehouse to Warehouse 4.0, human ability is needed for balance. Therefore, in the last three years, there have been many articles on new learning techniques and the development of new training programs on the required competencies in Industry 4.0 systems. Concerning new learning techniques, a key innovation is the use of virtual reality in the process of competence improvement by trainees. Examples of such articles are [175,176]. The latter also uses the concept of a digital twin. The curricula and training programs being developed deal with new technologies and their application to Warehouse 4.0 and intelligent manufacturing [177,178,179]. This increases participants’ interest in theoretical knowledge and encourages practical skills.

Meanwhile, the authors of [180] developed a project that intends to replicate the processes applied in a manufacturing factory, helping students to develop new technologies and solutions to real industry problems. New technologies also enable the creation of educational tools that reflect the natural working environment. An example of this is RobotAtFactory 4.0, presented by [181], which mimics a solution dedicated to a fully automated industrial logistics warehouse and helps identify related challenges in the execution of maintenance operations.

### 4.7. Method

A method is a particular procedure for accomplishing or approaching something, especially a systematic or established one. In this category, 59 articles were discovered and divided into 3 subcategories. 

The first subcategory is *algorithm*. In this subcategory, researchers analyzed algorithms whose task is to optimize the use of 4.0 technology and increase the efficiency of warehouse processes. Studies assigned to this subcategory concern:

- Algorithm for improving storage system [182,183,184,185,186,187];
- Algorithm for optimization routes or path planning of AGVs or multi-robot [188,189,190,191,192,193,194,195,196];
- Algorithm for optimization indoor positioning, tracking, or localization [197,198,199,200,201,202,203,204], and anti-collision algorithm for intelligent warehouse [205];
- Scheduling algorithm [206,207,208,209,210], and algorithm for task assignment [211,212,213,214,215,216,217,218];
- Algorithm to gain efficiency [219,220];
- Algorithm for developing 3D or dynamic environment [221,222,223,224].

The second subcategory is *data analysis*, which is those focused on studying data analysis approaches for decision making/support, such as:

- Proposing intelligent logistic inspection system based on big data [225];
- Analyzing big data for decision support [226], or big data warehouse [227] for decision making;
- Self-adapted SWARM Architecture [228];
- Analyzing data of predictive model for shipment delay and demand forecasting [229];
- Knowledge-based mining data analysis [230];
- Analyzing big data with a simulation model for a decision support system [231], or hybrid simulation model [232].

Other articles on methods, but not directly related to algorithms or data analysis, are classified in the subcategory *other*. The study that appeared in this group concerned:

- Developing versatile procedure for eliminating time waste in picking process [233]; method of digital transition [234];
- Developing intelligent logistic system based on ubiquitous information [235];
- Uses interval Type-2 Fuzzy approach for demand and order quantities with multi-objective vendor [236];
- Defining new control algorithm for real-time replenishment [237];
- Defining method of use of data to optimize lean manufacturing practices in the era of digitization and Industry 4.0 [238];
- Uses non-negative discriminative collective target nearest-neighbor representation algorithm for classifying data image [239].

### 4.8. Network

In Warehouse 4.0, many operations previously performed manually are supported by autonomous solutions such as AGVs (Automated Guided Vehicles), AMRs (Autonomous Mobile Robots), or UAVs (Unmanned Aerial Vehicles). These devices, in line with Industry 4.0, should be adequately connected to each other, and a properly prepared network of connections enables this. Extending the standard ETSI NFV reference architecture [240] proposed an implementation and orchestration mechanism to enable Cross-Slice Communication (CSC) in industrial environments. Experimental results using Warehouse Robotics show significant improvements in expected mission completion delay and transmission time using the proposed CSC approach compared to other cross-slice communication methods. This proposal applies to 5G (mobile communication) technology. In contrast, the results presented by [241] address a scenario moving toward 6G, where ultra-high performance backbone network slicing is associated with the part of an end-to-end solution to address ultra-reliable, low-latency communications for critical industrial operations for smart factories and manufacturing, intelligent warehouses and other Industry 4.0 applications and beyond.

### 4.9. Safety

Occupational health and safety are a significant part of all activities, including in the industrial world. Digital transformation and associated new technologies are bringing many changes to anthropotechnical systems, changing people’s working conditions and modifying the risks involved. Warehouse 4.0 operations increasingly use visual technology such as augmented reality, mixed reality, global vision, and virtual reality. An example of such a solution is the support of a picking worker through smart glasses to increase the efficiency of his work. However, it is necessary to observe that this could impair the comfort of his work and create occupational health and safety risks. For this reason, there are also publications on the assessment of this impact in the literature, such as the effects of smart glasses on eyesight [242], and visual and optometric issues of smart glasses [243].

### 4.10. Uncategorized

The uncategorized group has been supplemented with articles presenting research results unrelated to the distinguished areas because of the focus on another field instead of warehousing. Those articles focus more on industrial, mechanical design, aircraft management, etc. The warehouse appears in these publications as a testing ground, a link in the analyzed process, or possibly an area supporting manufacturing processes. Nevertheless, these articles were included in the analysis because they appeared in the search results and are related to Warehouse 4.0. The scope of the research included the following:

- The solution to extend the autonomy of machining centers by using a six-axis robot to replace the operator on work piece feeding operation [244];
- Implementing AR with gamification on order picking [245];
- Research about first report of driver injuries [246];
- Developing cost in logistics related industry 4.0 [247];
- Optimizing production related smart manufacturing process [248];
- Studying storage shelf deformation with FEA [249];
- Benchmarking of three low-cost and one medium-cost inertial analysis [250];
- Tracking asset and production [251];
- Create a system for localizing people being evacuated from a building when a disaster occurred in a workplace [252];
- Implementation RFID-based data for establishing inspection and maintenance interval of machine in production line [253];
- Big data analysis in EoL aircraft management [254];
- Trajectory planning for smart mobile robot [255];
- Designing model smart wearable devices for disabilities related to industry 4.0 [256];
- HMDFF (Heterogeneous Medical Data Fusion Framework) for medical data [257];
- Discover new algorithm for stress distribution [258];
- Designing automatic feed machine in fishponds [259];
- Single side priority-based algorithm for 3D printing center integration [260];
- Analyzing big data for risk management [261];
- Innovating operation exhibition of e-commerce by internet celebrity [262];
- Framework of a heterogenous multi-modal medical data fusion [263];
- Assessment of safety culture in major hazard industries [264].

## 5. Discussion

The development of technologies supporting Industry 4.0 systems has accelerated digital transformation in both manufacturing and logistics processes. This has significantly affected the technological transformation of peri-manufacturing systems, such as warehousing and internal logistics. This trend has caused more and more research on Industry 4.0 to focus on aspects of Manufacturing 4.0 and Warehouse 4.0. Strong trends toward developing digital supply chains also support implementing new digital solutions and 4.0 technologies in warehouses. These chains are oriented toward implementing techniques to improve their information exchange and supply chain management’s transparency, reliability, traceability, and efficiency [7]. It is impossible to achieve these goals without digitizing warehouse operations. 

### 5.1. Analysis of the Obtained Results

Our literature review highlighted 10 leading research areas and 24 subcategories. It should be noted that in most of these studies, the authors’ attention was primarily focused on the implementation of (1) Industry 4.0 technological solutions, such as IoT, augmented reality, RFID, visual technology, and other emerging technologies; and (2) autonomous and automated vehicles, such as automated/autonomous guided vehicles, autonomous robots, drones, etc., in warehouse operations processes. The presented research results reveal the challenges of implementing Industry 4.0 solutions in warehouse processes (Section 4.3 and Section 4.5) define the framework for the implementation and operation of these systems (Section 4.4), and opportunities are sought to optimize or increase the efficiency of warehouse processes implemented using new technologies (Section 4.3 and Section 4.7). At the same time, it should be emphasized that the scope of research related to the operation of smart warehouses is very comprehensive, as evidenced by the many highlighted primary categories and subcategories and the many individual publications included in the “Uncategorized” group. Some of these research areas are already well recognized, as evidenced by the large number of publications within a given subcategory and even the occurrence of review articles on a given topic. Examples of such research areas are IoT implementation (review articles) [36,37,38,39], augmented reality (review articles [26,27,28,29]), and other emerging technologies (review articles [30,31,32,33,34,35]). However, the large number of individual articles in the “Uncategorized” group indicates that many topics related to smart warehouses are only in the early stages of research. Such a conclusion is justified. The first period of development of the Industry 4.0 concept was focused primarily on the digitization and automation of manufacturing processes. Only their digitization forced changes in the next stage of the transformation and also in the processes supporting production systems, including warehouses. The COVID-19 pandemic has also reinforced the need for digitization and automation in supply chains [265]. The disruption resulting from high worker absenteeism has forced organizational and technological changes in the current implementation of warehouse operations. As a result, it can be expected that the number of publications on smart warehouses will continue to increase in the coming years.

It should also be noted that most articles focus primarily on the benefits of implementing Industry 4.0 solutions in logistics processes. The implemented technologies may be a way to eliminate human errors occurring in traditional warehouses, standardize processes and operations, improve the efficiency and effectiveness of operations, and improve communication and information exchange between the cooperating links of the supply chain [266]. In [267], the author also emphasizes that Industry 4.0 significantly improves sustainable practices if an organization focuses on humans. However, Bai et al. [268] indicate that autonomous robots, big data, blockchain, sensors, and analytics can positively influence the organization’s environmental sustainability practices.

However, because Warehouse 4.0 promotes a new approach to cargo handling in internal logistics, it is worth paying attention to the existing barriers to its implementation. Matt et al. [3] distinguished four groups of obstacles related to implementing Industry 4.0 solutions in the internal processes of enterprises. These are organizational, market, institutional, social, and ethical barriers. From the point of view of digital transformation in warehouses, organizational barriers highlighted in [3] should be considered critical, among which it is worth pointing out:

1. A skeptical attitude toward the advantages envisaged by a digitalized industry.
2. Lack of commitment and motivation within the company.
3. Substantial implementation and opportunity costs of integrating digital systems into existing IT solutions and databases.

Particularly noteworthy are the barriers to implementing Industry 4.0 solutions in the warehouse, highlighted by Kumar et al. [269]. The authors identified 17 such barriers and limitations in their research. Some of them coincide with the results presented by Matt et al. In this case, it is worth highlighting additionally [269]: (1) long return on investment; (2) high market competition and uncertainty; (3) lack of dynamic environment/systems and scalability; (4) lack of technical knowledge and support; (5) lack of skilled manpower; (6) lack of physical infrastructure; (7) lack of IT infrastructure; (8) lack of financial support. Analyzing existing limitations and barriers should be the starting point for the designed and implemented solutions. For this reason, these factors need to be considered, particularly in publications belonging to groups 4.3, 4.4, and 4.5.

### 5.2. Identification of the Research Gap

Our literature review allowed us to identify the research gaps that still exist, even though, as we have highlighted, the area of current research related to warehouses operating according to the 4.0 concept is broad. First, the small number of publications assessing the risks in smart warehouses is surprising. Only two articles dealt with the risks associated with using augmented reality and its impact on the health and safety of workers (Section 4.9). However, Industry 4.0 technologies also generate other risks for workers and various adverse events that can disrupt the warehouse’s product and material flows. This aspect should be the subject of detailed research since smart warehouse safety and efficiency depend on the enterprise’s risk management system. We considered the second research gap to be the lack of publications related to the maintenance of technical systems in Warehouse 4.0. One can find publications on the maintenance of Industry 4.0 systems in the literature—an example is a review article [270]. However, our search found no publication on maintaining specific 4.0 systems in the warehouse area. There is also a lack of studies on using 4.0 technologies in warehouse maintenance processes (e.g., augmented reality and virtual reality in increasing maintenance operations’ efficiency and the maintenance staff’s competence).

### 5.3. Summary of the Discussion

Summarizing the results of our research, it is important to indicate the novelty of the results obtained. They can be described in terms of three main achievements:

1. General nature of the conducted literature review.

In Section 4.1, we presented a compilation of review articles related to the operation of warehouses in the era of Industry 4.0. Most of these papers presented results focused on a specific research problem—applying specific technological solutions (Internet of Things, emerging technologies, augmented reality, artificial intelligence). On the other hand, in these reviews, the smart warehouse appeared only as one element of a broader research area (supply chain management, improvement in industry 4.0, impact of industry 4.0 on logistics). Our research focused on articles describing the functioning of smart warehouses, and the scope of the proposed classification framework covers all aspects related to the design, operation, and maintenance of Warehouse 4.0, which have been the subject of publications so far.

2. Identification of two research gaps in the analyzed literature review

The critical analysis of the literature made it possible to identify significant research gaps from the point of view of further development of the Warehouse 4.0 concept and the reported demand for scientific analyses. From the point of view of our further activities, we have identified two critical research gaps regarding the lack of studies on (1) risk assessment and management in warehouses, taking into account specific operating conditions related to the Industry 4.0 environment; and (2) selection of maintenance strategies for technical systems supporting logistics processes in Warehouse 4.0.

3. Development of a new classification framework for Warehouse 4.0 publications.

Our framework for classifying research related to Industry 4.0 in magazines covers a wide range of topics. Despite this, we still see the potential for further development in these areas, also based on the identified research gaps. For this reason, in the next step, it is desirable to publish research results that will allow us to supplement our framework with two additional basic categories, namely:

(a) “Risk assessment”, which should consider security issues (cyber security, employee health, and life) and disruptions affecting the logistics service level provided by the warehouse.
(b) “Maintaining smart warehouses”, which should include selecting appropriate maintenance strategies for modern technical systems, using digital technologies in maintenance processes, changing the requirements, and improving the competence of maintenance staff.

Our future activities aim to achieve results that fit into both of the above categories.

## 6. Conclusions and Future Work

The analyses presented in the article concerned the group of 249 publications identified in a research investigation using the PRISMA method. All of these publications dealt directly or indirectly with the warehouse operation concerning the concept of Industry 4.0. The results presented in the article consider the wide range of research in this area. As a result, they can be used by both theoreticians and practitioners. The classification framework we have proposed represents the most important directions of research work published over the past 5 years in journals worldwide. This enables industry representatives to gain knowledge of the leading technologies used in smart warehouses and the challenges and good practices associated with their implementation. The classification procedure carried out in the article points them to specific documents they can use to acquire this knowledge. At the same time, the results of our research indicate to theorists the dominant directions of research work that have been undertaken in recent years. The research gaps identified, on the other hand, indicate to them the need for the results of future analyses, the fulfilling of which will allow them to improve the operation of Warehouse 4.0.

The five-stage research procedure we adopted was comprehensive. We used a recognized journal database that registers high-profile and peer-reviewed publications to search for documents in line with the stated goal. In this way, we ensured the documents accepted for analysis were high quality. Our proposed selection of keywords to search for relevant records was preceded by a thorough analysis of review articles in the area. However, the focus of attention only on articles and proceedings papers, and the lack of non-reviewed documents in the analysis, which often appear in magazines and on industry websites, can be considered a limitation. However, such action is justified by our care for the quality of published results accepted for analysis. The selection of keywords and the type of documents also resulted in only English-language documents being indicated for analysis by the search engine. Consequently, the study did not include documents prepared in other language versions. However, it should be emphasized that their absence from the identified group means that such publications did not even have an English-language version of the title, abstract, and keywords.

The results in the article allow us to answer all the research questions posed in the Introduction. From the point of view of further work, it is important to answer question Q3, which allowed us to identify the existing research gaps. Our future research will therefore focus on assessing risk factors, the effects of adverse events, and methods of risk analysis for improving the current operation of intelligent warehouses. On the other hand, the second direction of our work will be the development of maintenance strategies for technical systems that support goods flows in Warehouse 4.0.

## Figures and Tables

**Table 1:** Research area.

Research Area	Number of Publications	Percent (%)
Automation & Control System	58	23.3%
Business & Economics	55	22.1%
Computer Science	30	12.0%
Education & Educational Research	23	9.2%
Energy & Fuels	20	8.0%
Engineering	15	6.0%
Instrument & Instrumentation	11	4.4%
Material Science	9	3.6%
Operation Research & Management	8	3.2%
Remote Sensing	5	2.0%
Robotics	5	2.0%
Science & Technology	3	1.2%
Social Science	3	1.2%
Telecommunication	2	0.8%
Transportation	2	0.8%
**Total Articles**	**249**	**100%**

**Table 2:** Articles belonging to category “Implementation”, source: own work.

Emerging Technology	Articles
**Leading Emerging Technologies**	
Augmented Reality	[142,143,144]
Internet of Things	[1,13,145,146,147,148,149,150,151,152]
RFID	[153,154,155,156,157,158,159,160]
Visual Technology	[161,162,163,164]
**Other Emerging Technologies**	
Ultra-Wideband	[165]
Platform	[166]
Machine Learning	[167,168]
Autonomous Vehicle	[169]
Real-Time Location System	[170]
Shuttle	[171]
Blockchain	[172]
Digital Twin	[173]
Digitalization Work Environment	[174]

## Data Availability

Not applicable.

## Supplementary Materials

The following supporting information can be downloaded at: https://www.mdpi.com/article/10.3390/s23084105/s1.
