# Supplementary material for: Intelligent Warehouse in Industry 4.0—Systematic Literature Review
Source: Sensors (Basel). 2023 Apr 19;23(8):4105. doi: 10.3390/s23084105 (PMC10146052; doi:10.3390/s23084105)
Supplement: Supplementary file 1 [file sensors-23-04105-s001.zip › sensors-2305778-supplementary.pdf]

Table S1. Category and subcategory of articles.

Category	Subcategory	Article
**Literature Review**	Artificial Intelligent	(Ellefsen et al., 2019; Khalifa et al., 2021; Wachnik, 2022)
	Augmented Reality	(Husár & Knapčíková, 2021; Lagorio et al., 2022; Plakas et al., 2020; Sidiropoulos et al., 2021)
	Emerging Technology	(Brunetti et al., 2022; Domański, 2019; Ilin et al., 2019; Jagtap et al., 2020; Perotti et al., 2022; Żuchowski, 2022)
	IoT	(Fatima et al., 2022; Kho & Aknin, 2020; Ponis & Efthymiou, 2020; Rejeb et al., 2019)
	Manufacturing	(Ammar et al., 2021; Erdei et al., 2018; Vlachos et al., 2022)
	Storage System	(Cinar & Zeeshan, 2022; Edouard et al., 2022; Pahl, 2019)
	Supply Chain Management	(A. Choudhury et al., 2021; Chupanova et al., 2021; Henao-Hernández et al., 2021; Nguyen & Nguyen, 2019)
	Other	(Agalianos et al., 2020; Behera et al., 2018; Dantas & Barbalho, 2021; Dobos et al., 2021; Dolgui & Ivanov, 2022; El Hamdi & Abouabdellah, 2022; Ezzy et al., 2020; Forcina & Falcone, 2021; Harrison, 2019; Jepsen et al., 2020; Klumpp et al., 2019; Kurschl et al., 2021; X. Liu et al., 2018; Maniah & Milwandhari, 2020; Mao et al., 2018; Mouschoutzi & Ponis, 2022; Puneet et al., 2021; Puviyarasu & Cunha, 2021; B. D. Sarkar et al., 2023; Song et al., 2019; Tubis & Poturaj, 2021; van Geest et al., 2021b)
**Assessment/Evaluation**	Case Study	(Abideen & Mohamad, 2021; Binar et al., 2022; Costa & Santos, 2018; Fernández-Caramés et al., 2019; Kattepur, 2019; Kattepur, Mukherjee, et al., 2018; Keivanpour, 2022; Motroni et al., 2022; Murauer & Pflanz, 2018; Nunes & Barbosa, 2020; Prabhu & Chowdhury, 2021; Zhou et al., 2019)
	Maturity Model	(Alhazred & Dee, 2018; Bastos Porsani et al., 2021; Zoubek et al., 2022; Zoubek & Michal, 2020; Zoubek & Simon, 2021b)
	Other	(Ridolfi et al., 2019; Ud Din et al., 2021; Val et al., 2020)
**Design/Model**	Algorithm	(Dharmasiri et al., 2020; Farajzadeh et al., 2020; X. He & Prabhu, 2022; Palominos et al., 2019; Sahlab et al., 2021; Thanh, 2022; Witczak et al., 2019; Y. Ekren & Arslan, 2022; Zhai et al., 2022)
	Layout	(Chaudhuri et al., 2019; El Ouadaa et al., 2018; Tiefeng et al., 2020; H. Zhang et al., 2019)
	Tool/System	(A et al., 2022; Alajami et al., 2022; Asghar Khan et al., 2021; Balaska et al., 2022; D'Souza et al., 2020; Fernandes et al., 2019; Forte et al., 2022; F. Gao & Cheng, 2020; Grecuccio et al., 2020; Huo et al., 2020; Kattepur, Dey, et al., 2018; Kihel et al., 2022; Konstantinidis et al., 2022; Lian et al., 2022; M. A. Limeira et al., 2019; Lin et al., 2021; Lorenc & Lerher, 2020; Lototsky et al., 2019; Panigrahi et al., 2019; Rocha et al., 2022; H. Sun et al., 2020; Trstenjak et al., 2022; van Geest et al., 2021a; Vitolo et al., 2022; Xi-Kun et al., 2020; Y. Zhang & Pan, 2022)
	Other	(Gong et al., 2019; Sevic & Keller, 2021)
**Framework**		(Campos et al., 2019; S. Choudhury et al., 2019; Coito et al., 2019; Din et al., 2018; Kattepur & P, 2019; H. Ma, 2019; Mostafa et al., 2019; Nantee

		& Sureeyatanapas, 2021; Sharma et al., 2022; Stetter et al., 2021; Winkelhaus et al., 2021; Yavas & Ozkan-Ozen, 2020; Zoubek & Simon, 2021a)
**Implementation**	Augmented Reality	(Madeira et al., 2021; Papcun et al., 2019; Piardi et al., 2019)
	IoT	(Benzi et al., 2019; Boppana & Bagade, 2022; Garrido-Hidalgo et al., 2019; Hamdy et al., 2022; Keung et al., 2020; Khan et al., 2022; Lee et al., 2018; Maheshwari et al., 2021; Yanabe et al., 2020; R. Zhang et al., 2022)
	RFID	(Braglia et al., 2019; X. Chen, Huang, et al., 2018; X. Chen, Wei, et al., 2018; D'Avella et al., 2022; Fontaine et al., 2021; Kokkonen et al., 2022; Motroni, Buffi, Nepa, et al., 2021; Tripicchio, Unetti, et al., 2022)
	Visual Technology	(Tang & Zeng, 2021; Vukicevic et al., 2021; Yin et al., 2022; Zheng & Lu, 2022)
	Other	(Barral et al., 2019; Coito et al., 2020; Ferrari et al., 2022; Fontaine et al., 2020; Gružauskas et al., 2018; Halawa et al., 2020; Lerher, 2018; Shahbazi & Byun, 2020; Smajic & Bosco, 2021; Vashist et al., 2022)
**Improving Knowledge**		(Braun et al., 2022; D. He, 2022; Jena et al., 2022; Osorio-Oliveros et al., 2022; Pan et al., 2022; Verner et al., 2020; S. Wang et al., 2021)
**Method**	Algorithm	(Borisoglebskaya et al., 2019; H. Chen et al., 2018; M. Chen et al., 2021; Dundar, 2021; el Hamdi et al., 2020; El Hamdi et al., 2020; Fu et al., 2020; J. Gao et al., 2022; Guo et al., 2022; Han et al., 2022; Jin & Yu, 2022; Kattepur, Rath, et al., 2018; H. Li et al., 2022; Z. Li et al., 2020; M. Limeira et al., 2021; M. Liu et al., 2022; Y. Liu et al., 2019, 2022; J. Ma et al., 2022; Y. Ma, 2021; Martins et al., 2019; Mo & Li, 2019; Motroni, Buffi, & Nepa, 2021; Moura et al., 2021; Nguyen Duc et al., 2020; Novak et al., 2020; Oliveira et al., 2022; Oxenstierna et al., 2022; Qin et al., 2022; Qiu et al., 2019; Rey et al., 2021; C. Sarkar et al., 2018; Souto et al., 2021; Y. Sun & Li, 2020; Tripicchio, D'Avella, et al., 2022; Tu et al., 2021; Z. Wang et al., 2019; Wu et al., 2019; Xiong et al., 2022; Xue et al., 2019; Q. Yang et al., 2022; S. Yang et al., 2021; Yetkin Ekren, 2021; Zoubek et al., 2021)
	Data Analysis	(X. Chen, 2022; Galvão et al., 2022; Guerreiro et al., 2019; Keung et al., 2021; Silva et al., 2021; Sternberg & Atzmueller, 2018; Vieira et al., 2019a, 2019b)
	Other	(Ashraf & Shahid, 2021; Bevilacqua et al., 2019; Duca et al., 2020; H. He et al., 2021; Kihel, 2022; Omar & Plapper, 2019; Y. Sun et al., 2022)
**Network**		(Dimolitsas et al., 2021; Ricart-Sanchez et al., 2021)
**Safety**		(Herzog & Beharic, 2020; Vujica Herzog et al., 2018)
**Uncategorized**		(Barbosa et al., 2018; Bright & Ponis, 2021; Bunn et al., 2022; Čámská & Klečka, 2020; Chakroun et al., 2022; D.-C. Chen & Kuo, 2018; Cramer et al., 2019; Frankó et al., 2020; Janczak et al., 2022; Karwasz & Paczesny, 2021; Keivanpour & Kadi, 2018; Lee et al., 2019; Periša et al., 2021; Ren et al., 2021; Tabatabaei et al., 2021; Tjhin & Riantini, 2021; Tuzkaya & Sahin, 2021; Vieira et al., 2020; J. Zhang & Zhao, 2019; Y. Zhang et al., 2022; Zwetsloot et al., 2020)
